# Use of the Image Guided Minimally Invasive BrainPath System to Evacuate Spontaneous Cerebellar Hemorrhages

**DOI:** 10.7759/cureus.16124

**Published:** 2021-07-02

**Authors:** Kainaat Javed, Mousa K Hamad, Ryan Holland, Adisson N Fortunel, Adam Ammar, Phillip C Cezayirli, Neil Haranhalli, David J Altschul

**Affiliations:** 1 Neurological Surgery, Montefiore Medical Center, Moses Campus, New York, USA

**Keywords:** minimally invasive, hematoma evacuation, infratentorial, brainpath, craniotomy

## Abstract

BrainPath (NICO, Indianapolis, Indiana) is a tool that can be used to evacuate supratentorial hematomas due to spontaneous intracerebral hemorrhage (ICH). However, when ICH occurs in the posterior fossa, an open approach is often undertaken to evacuate the hematoma. The application of minimally invasive technology, while available, has not been well established. Our objective was to describe the use of the image-guided, minimally invasive BrainPath system to evacuate a spontaneous cerebellar hemorrhage. We present the case of a sixty-four-year-old male patient with a cerebellar hematoma due to hypertensive hemorrhage. The patient's medical record, including the history and physical, progress notes, operative notes, discharge summary, and imaging studies were reviewed to document the clinical presentation as well as the details of the operative technique and postoperative outcomes in this paper. We discuss the technical nuances of the operative points in detail. In our example case, the BrainPath system was successfully used to evacuate the cerebellar hematoma and no procedural-related complications occurred. The patient's recovery remained uncomplicated at three months of follow-up. In summary, the BrainPath system offers a less invasive alternative to open evacuation for cerebellar bleeds.

## Introduction

There has been renewed interest in the surgical management of intracerebral hemorrhage (ICH) using minimally invasive approaches. Although the results from the minimally invasive surgery plus recombinant tissue plasminogen activator (rt-PA) for ICH evacuation phase III (MISTIE-III) trial showed no functional benefit to surgical intervention, subgroup analyses did show a potential subset of patients that might have improvement with surgical evacuation [1]. The MISTIE technique is somewhat cumbersome given the requirement for injection of tissue plasminogen activator (tPA) post-procedure. Other more reasonable minimally invasive techniques such as image-guided endoscopic techniques and exoscopic-guided hematoma evacuation may achieve more immediate results with a similarly less invasive approach, without the requirement of a long-term catheter in the clot cavity. The BrainPath (NICO, Indianapolis, IN) system is one example of a technique that utilizes an exoscope or microscope and tubular retraction system to evacuate deep-seated hematomas with minimal trauma to white matter tracts. The use of the BrainPath system has been described in the literature and is shown to be safe, effective, and associated with functional independence [2].

The American Heart Association recommends surgical intervention for cerebellar hemorrhages that are greater than three centimeters (cm) in diameter, especially if they are associated with obstructive hydrocephalus [3]. Cerebellar hematomas are traditionally evacuated via an open approach and include bony decompression to reduce the risk of secondary injury or swelling leading to brainstem compression [4]. Three papers published recently discuss the application of minimally invasive technology to control cerebellar hemorrhages. One paper discusses a technique that consists of clot aspiration via mini-craniectomy [5] while the other two papers describe an endoscopic technique using stereotactic navigation [6,7]. To our knowledge, the use of the exoscopic BrainPath system for a cerebellar hematoma evacuation has not been previously described in the literature.

## Technical report

Operative technique

Operating Planning, Anesthesia & Patient Positioning

**Figure 1:**
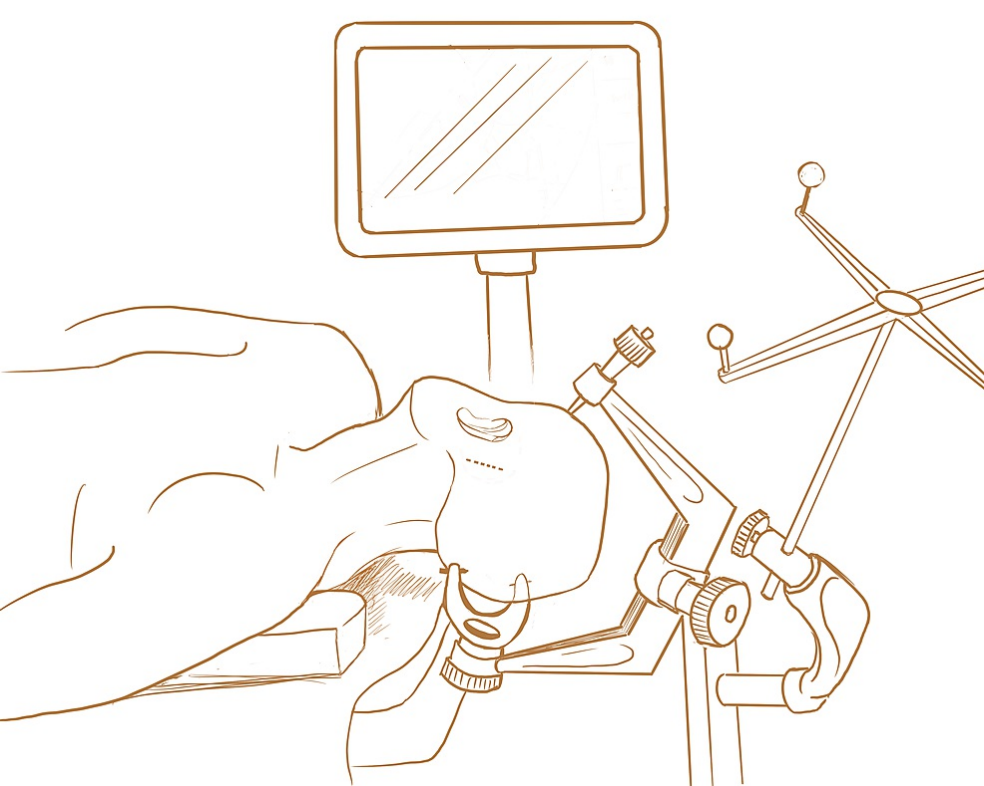
Patient Positioning Patient is positioned supine with head turned to the contralateral side. Shoulder roll is placed beneath ipsilateral shoulder. Patient is pinned to the Mayfield Head Frame. Dashed line behind left ear indicates site of skin incision. Stereotactic navigation is set up prior to the start of the operation.

Informed consent should be obtained from the patient or their family before the procedure. The patient is sedated and intubated by the anesthesiology team. A Foley catheter is inserted. The patient's eyes are taped shut after ointment is applied to prevent corneal abrasion. A Bair HuggerTM is placed over the exposed lower body to maintain control of core body temperature. Sequential compression devices (SCD) are used for deep venous thrombosis (DVT) prophylaxis. The patient is placed supine with the head turned to ninety degrees so the side of the hematoma is facing superior. A shoulder roll is placed underneath the ipsilateral shoulder. The patient is placed in a Mayfield head frame (Figure 1).

Retrosigmoid Craniectomy

The patient is prepped and draped in a standard sterile technique. The patient is given prophylactic antibiotics. The patient is registered to a Computed Tomography (CT) scan (that was obtained pre-operatively) using surface landmarks on the BrainLab navigation system to an accuracy within two millimeters. A linear, retrosigmoid paramedian incision is planned around the planned entry site on the left or right side depending on the laterality of the bleed. The transverse sinus and sigmoid sinus should be identified and marked. 

**Figure 2:**
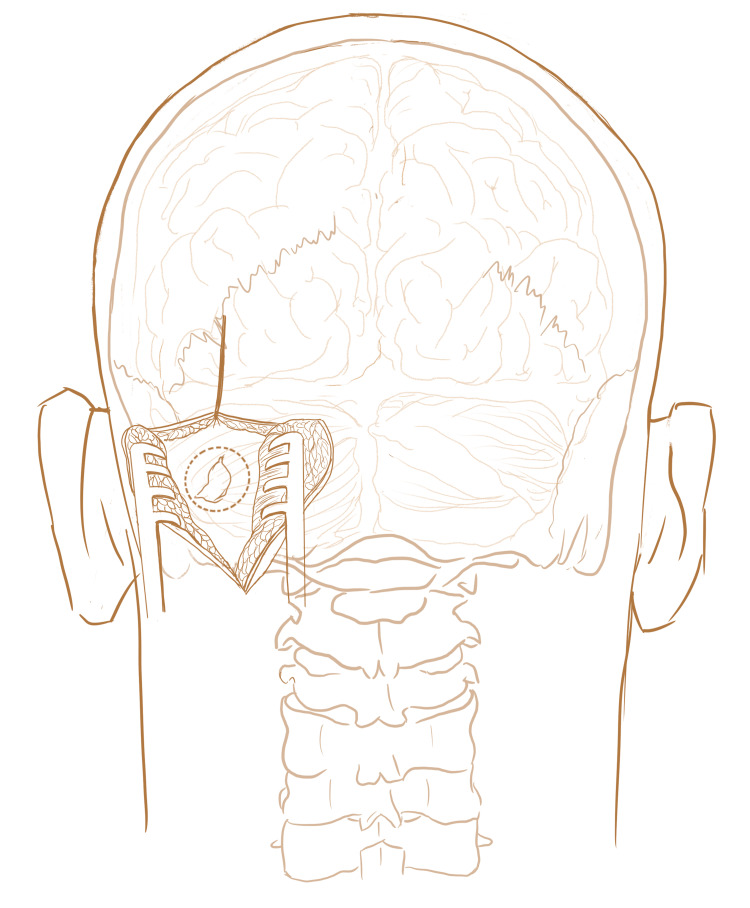
Retrosigmoid Craniectomy Location of the cerebellar hematoma is shown. Dashed line indicates where the bone will be drilled for a retrosigmoid craniectomy.

Using a #10 scalpel, the skin is incised to the level of the periosteum. The scalp is dissected together from the periosteum and retracted forward using cerebellar retractors. Of note, if using an electromagnetic navigation system such as the Axiem (Medtronic, Minneapolis, Minnesota) then you should avoid using cerebellar retractors and use skin hooks instead as the metal from the retractor can interfere with the navigation system. Then, using a perforator drill, two burr holes are placed and a craniotomy flap is completed using a combination of a B1, and M33 for widening the craniotomy if needed. In general, the craniotomy flap can be small but needs to be large enough to be able to angle the BrainPath system in the necessary trajectory to reach the clot cavity (Figure 2).

Using the BrainPath System

**Figure 3:**
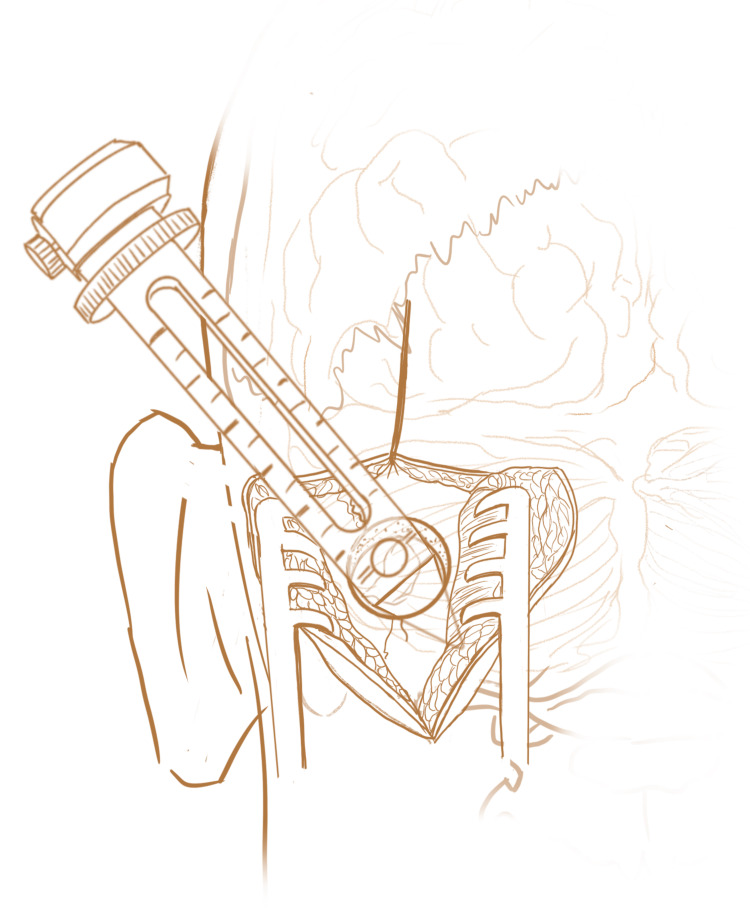
Use of the BrainPath System The BrainPath system is being inserted through the site of the retrosigmoid craniectomy and will be used to evacuate the cerebellar hematoma using a combination of suction, aspiration and the Myriad device.

The dura is coagulated using a bipolar cautery, incised using a #11 blade, and then cut in a stellate fashion using Metzenbaum scissors. The chosen sulcus is identified and carefully dissected apart. Then using BrainLab navigation, the BrainPath NICO system is placed into the intracerebral hematoma under direct navigational assistance and the Myriad device (NICO Corp.) is used for suction and aspiration to remove the residual clot (Figure 3). A microscope or exoscope can be draped and used for visualization of the clot removal. This fluid sample may be sent to pathology as a permanent specimen. Meticulous hemostasis is achieved using a combination of bipolar electrocautery and hemostatic agents. 

Closing & Procedure Wrap Up

The dura is closed using 4-0 nurolon sutures. Bone saved from the craniotomy may be replaced, a titanium mesh may be placed or a craniectomy defect may be left depending on the size of the hemorrhage, and degree of anticipated swelling. Further bony decompression of the foramen magnum can be done to decrease the risk of tonsillar herniation from secondary swelling. The galea is closed using 2-0 vicryl sutures. The skin is closed using interrupted, vertical mattress stitches with 3-0 nylon sutures. All needle counts, sponge counts, & instrument counts should be correct at the end of the case.

Illustrative Case

**Figure 4:**
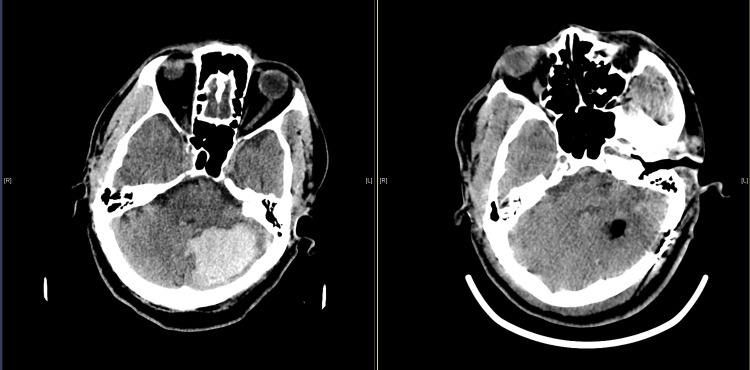
Pre and Post Operative CT Scan Pre-operative and Post-Operative CT scans of our patient are shown for a side by side comparison.

A 64-year-old male with a past medical history of hypertension who was not on any medication presented to the Emergency Room (ER) via Emergency Medical Services (EMS). The patient was found confused and without his garments in his bathroom by his family one day prior. When his confusion continued to worsen over the course of the day, his family ultimately called EMS. Physical examination was notable for inattention, left gaze palsy, and bilateral dysmetria. The patient's presenting blood pressure was 195/99. CT scan of the head showed a large, acute 25 cubic centimeter (cc) hemorrhage in the left cerebellum, causing obstructive hydrocephalus due to mass effect on the fourth ventricle along with signs of tonsillar herniation. There was also a small, 2 cc acute hemorrhage in the left posterior lateral thalamus. Etiology deemed to be hypertensive. His ICH score was two. The patient was taken to the operation room (OR) emergently for hematoma evacuation using the technique described in this paper. He was extubated and transferred to the neuroscience intensive care unit (NSICU) where he remained until postop day four. Postop CT scan showed expected surgical changes in the left cerebellum, persistently enlarged ventricles, and unchanged parenchymal hemorrhage in the left thalamus (Figure 4). The patient continued to receive serial CT scans in the NSICU and they were stable. Long-term electroencephalography (EEG) monitoring showed no seizure-like activity. On post-op day three, the patient was diagnosed with right lower lobe pneumonia on chest x-ray and started on a four-day course of ceftriaxone. He was transferred to the stroke service for further management and ultimately discharged to acute rehab. CT scan of the head performed just before discharge showed interval resolution of the left thalamic hemorrhage and decreased ventricular size. A telephone encounter near the three-month mark revealed that patient's modified Rankin score (mRS) at the time was two. 

## Discussion

The preoperative and postoperative considerations for this technique are similar to those for an open evacuation of a cerebellar hemorrhage. Preoperatively, the patient should undergo a computed tomography angiography (CTA) scan to identify patients at risk for hematoma expansion and to exclude any vascular malformations as the underlying cause of the hemorrhage. Postoperatively, patients should get a CT scan and should be treated with blood pressure management, anti-epileptics (AEDs), intracranial pressure (ICP) monitoring, and pain control [3]. Complications to look out for include rebleeding, perihematomal edema, hydrocephalus, seizures, venous thrombosis, fever, and infections [8].

One indication for this technique may be patients who cannot be placed in the prone position for an extended period of time. When used for posterior fossa surgery, the prone position is associated with adverse effects such as brachial plexus injury, paraplegia, air embolism, etc. [9-11]. In this technique, the patient is positioned supine with the head turned to the side, thus theoretically decreasing the risk for these adverse events. This technique would likely be contraindicated in patients who present with a low Glasgow Coma Scale (GCS) score (<8), large hemorrhage volume (>30 cc), significant hydrocephalus, or signs of tonsillar herniation. These patients are in urgent need of an external ventricular drain (EVD) placement or a suboccipital craniectomy and are not good candidates for this technique.

The technique described in this paper regarding the use of the BrainPath system to evacuate cerebellar hematomas is not different from the technique used to evacuate supratentorial hematomas using this technology. The steps involved and the actual use of the BrainPath system is identical. EVD placement depends on the presenting neurological exam and imaging and is up to the discretion of the treating neurosurgeon. The only difference lies in the type of craniotomy performed. In this technique, we performed a retrosigmoid craniotomy to gain direct access to the hematoma location. The retrosigmoid craniotomy is also known as "the keyhole craniotomy" because it is a minimally invasive approach that provides safe and easy access to the brainstem and cerebellum [12]. In this case, we performed a retrosigmoid craniectomy in order to compensate for any potential secondary swelling that may occur.

It is easy to compare the benefits of using this technique to evacuate a cerebellar hematoma over a larger craniectomy and evacuation given its precision and minimally invasive nature. However, other minimally invasive techniques such as the ones discussed by Kellner et al. and Atsumi et al. have been documented. Kellner et al. describe a minimally invasive approach using a mini-craniectomy that is done in the prone position without the use of stereotactic navigation [5]. Atsumi et al. describe a minimally invasive endoscopic approach that seems superior to traditional craniectomy [6]. However, an exoscopic tubular retraction system such as BrainPath allows for greater working room, enables the surgeon to use a microscope or exoscope depending on their preference, and leads to automated hematoma evacuation and preservation of the surrounding brain [13]. A larger corridor may assist in achieving hemostasis compared to the smaller corridor from endoscope alone techniques.

## Conclusions

The BrainPath (NICO, Indianapolis, Indiana) system is an image-guided, minimally invasive system that uses an exoscope and tubular retraction to remove intracerebral hematomas, tumors, and other deep lesions in the brain with the goal of reducing the amount of trauma to normal brain and white matter tracts. The BrainPath system has been used to successfully evacuate supratentorial hemorrhages and is well described. In this technical report, we describe an operative technique that can be used to apply this technology to spontaneous cerebellar hemorrhages. The patient discussed in this case experienced no procedural complications and had a good outcome. That being said, further studies are warranted to assess the safety and efficacy of this novel technique. 
